# Tumor Necrosis Factor Alpha -308 G/A Single Nucleotide Polymorphism and Risk of Sperm Abnormalities in Iranian Males

**DOI:** 10.22074/ijfs.2017.4830

**Published:** 2017-02-16

**Authors:** Maryam Khademi Bami, Masoud Dehghan Tezerjani, Fateme Montazeri, Hamid Reza Ashrafzadeh Mehrjardi, Saeed Ghasemi-Esmailabad, Mohammad Hasan Sheikhha, Seyed Mehdi Kalantar

**Affiliations:** 1 Medical Biotechnology Research Center, Ashkezar Branch, Islamic Azad University, Ashkezar, Yazd, Iran; 2 Genetic Unit, Research and Clinical Center for Infertility, Shahid Sadoughi University of Medical Sciences, Yazd, Iran

**Keywords:** Infertility, Cytokines, Tumor Necrosis Factor Alpha, Polymorphism, Restriction Fragment Length Polymorphism

## Abstract

**Background:**

Signaling molecules such as cytokines regulate spermatogenesis during the maturation of germ cells and sperm apoptosis. Tumor necrosis factor alpha (TNFα) is one of the most-documented cytokines that is involved in spermatogenesis. We investigated the association of the TNFα -308 G/A single nucleotide polymorphism with sperm abnormalities in Iranian males.

**Materials and Methods:**

This case-control study included 180 infertile men who re- ferred to Yazd Research and Clinical Center for Infertility and 100 healthy normospermic controls. Infertile men were classified into four groups of azoospermia (n=91), oligospermia (n=26), teratospermia (n=30) and asthenoteratospermia (n=33). After sperm analysis, DNA was extracted from blood and polymerase chain reaction-restriction fragment length polymorphism (PCR-RFLP) was carried out for the genotyping of TNFα- 308 G/A.

**Results:**

The A allele was significantly associated with sperm abnormality in our population [(P<0.001, odds ratios (OR) 95% confidence interval (CI)=2.31]. In addition, the A allele was also associated with azoospermia (P<0.001, OR (95% CI)=2.484), oligospermia (P=0.005, OR (95% CI)=2.51) and teratospemia (P<0.001, OR (95% CI)=3.385) but not with asthenoteratospermia (P=0.623).

**Conclusion:**

Our data suggest that this single nucleotide polymorphism (SNP) maybe associated with the risk of sperm abnormality in infertile men of Iranian origin.

## Introduction

It is estimated that about 15% of couples globally suffer from infertility. Male infertility constitutes 50% of causes among which genetic factors are mainly responsible (1, 2). Other causes of male infertility maybe related to post-testicular obstruction, endocrine dysfunction and vascular abnormalities (3). During the last decade, it has become clear that some signaling molecules that mediate the intercellular communication and integration have an important role in the hormonal regulation of germ cell maturation in testis. Cytokines are mediator molecules that are involved in this regulation and as a result have an important impact on spermatogenesis (4, 5). Among cytokines, tumor necrosis factor alpha (TNFα) is not only the most studied molecule but also the most potent in germ cell apoptosis, peritubular cell secretion and regulation of spermatogenesis (6). Its receptors are present in Sertoli and Leydig cells, allowing TNFα to regulate secretion from these cells (7). Some studies have shown a negative association of TNFα plasma levels with sperm motility and morphology (8, 9). The effect of TNFα on testosterone production, which has a direct impact on male infertility, has also been reported in some experimental models (10, 11).

The *TNFα* gene as a single copy gene is located on chromosome 6p21.3 within the major histocompatibility complex (MHC) gene cluster (12). Gene variation such as single nucleotide polymorphisms (SNPs) in *TNFα* gene can alter TNFα production. Several SNPs including -308 G/A, -1031 T/C, -863 C/A, -857 C/T, -575 G/A, -376 G/A, -244 G/A and -238 G/A in the promoter region of the gene have been investigated (13). The -308 G/A SNP in the promoter region of *TNFα* has been implicated to increase promoter activity, leading to an increased production of TNFα in blood (14, 15). Some studies have reported a negative association of TNFα with sperm motility and morphology (9, 16, 17). Zalata et al. (18) showed the association of the -308 G/A SNP with increased seminal caspase-9 and decreased sperm motility, count, morphology, acrosin activity and seminal a-glucosidase. Shukla et al. (19) showed that there is a strong association between this SNP and male infertility in the Indian populations of Uttar Pradesh.

In this study, given that TNFα is an important regulator of steroidogenesis and may affect spermatogenesis, we investigated the association of the TNFα -308 G/A SNP with different kinds of sperm abnormality in infertile males of Iranian origin.

## Materials and Methods

This case-control study included 180 infertile males as the case group and 100 healthy normospermic individuals as the control group. The case individuals were recruited from Yazd Research and Clinical Center for Infertility from September 2012 until August 2013. They were divided based on sperm abnormality into azoospermia (n=91, AZ group), oligospermia (n=26, OL group), teratospermia (n=30, T group) and asthenoteratospermia (n=33, AT group) groups. This study was approved by the Ethics Committee of Shahid Sadoughi University of Medical Sciences, Yazd, Iran. Written informed consent was obtained from each individual. All semen analysis and clinical examinations were done according to the World Health Organization guidelines (20).

### Tumor necrosis factor alpha -308 polymorphism genotyping

Genomic DNA was extracted from whole blood samples using the salting out method. We used the polymerase chain reaction-restriction fragment length polymorphism (PCR-RFLP) method for genotyping of TNFα -308G/A. The F: 5'-AGGCAATAGGTTTTGAGGGCCAT-3' and R: 5'-TCCTCCCTGCTCCGATTCCG-3' primers were used to amplify a107 bp fragment of the TNFα promoter that included this SNP. PCR was carried out in a total volume of 25 μl containing 3-5 μl genomic DNA, 1 μl of each primer (10 μM) and 12.5 μl of PCR Master Mix (Cinnagen, Iran) and dH2O. The condition of DNA amplification was an initial denaturation step at 94°C for 5 minutes, followed by 35 cycles of 94°C for 40 seconds, 60°C for 1 minute and 72°C for 40 seconds, and a final extension step at 72°C for 5 minutes and hold at 4°C. Subsequently, the PCR products were digested with NcoI restriction enzyme (14 hours at 37°C) and the specific bands were identified using 2% agarose gel electrophoresis in 1X Tris/Borate/EDTA (TBE) buffer and visualized under the ultraviolet (UV) light. When digested, the PCR fragment was cleaved into two fragments with sizes 87 bp and 20 bp.

### Statistical analysis

The frequency of alleles and genotypes were compared with a 2×2 contingency table using Chisquared and Fisher’s exact test. Fisher’s exact test was used when sample sizes were small in each category. We considered P<0.05 as a statistically significant and 95% confidence interval (CI) for calculating odds ratios (OR).We used the SPSS statistical software (version 20, SPSS Inc., Chicago, IL, USA) for all statistical analyses.

## Results

**Fig.1:**
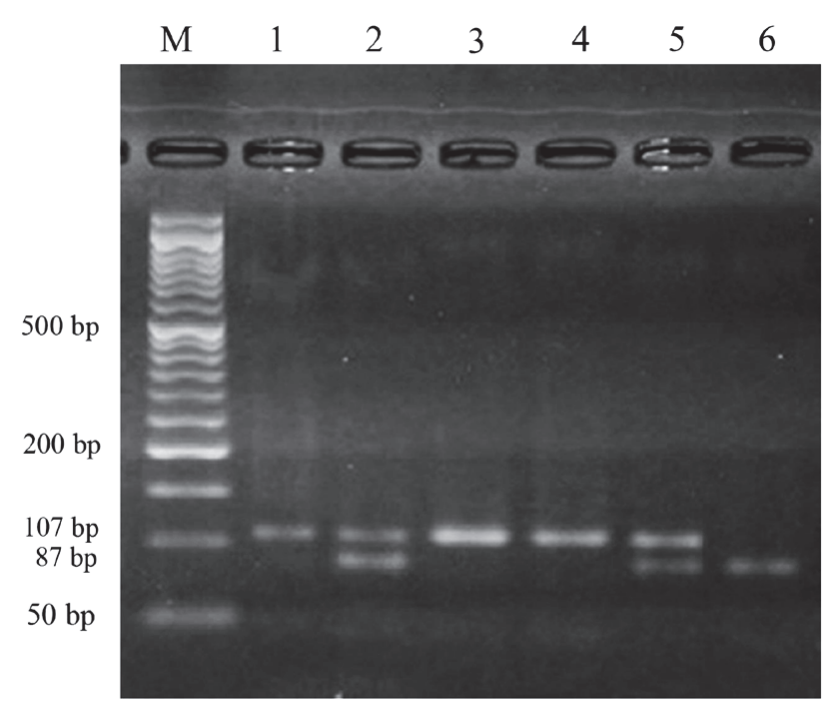
The results of polymerase chain reaction-restriction fragment length polymorphism (PCR-RFLP) analysis of TNFα -308 polymorphism. Lane M shows the molecular weight marker, Lanes 1, 3 and 4 show the AA genotype. Lanes 2 and 5 show the GA genotype. Lane 6 shows the GG genotype.

In this study, PCR-RFLP was able to identify both alleles efficiently at position -308 in the promoter region of *TNFα* gene (Fig .1).

Table 1 shows the related parameters of each group. The frequencies of alleles and genotypes and their association with the studied group are listed in Table 2. Ancestral genotype GG and allele G were taken as reference. Statistical analysis showed that there is a significant association between this SNP and the AZ, O and T patient groups but not with the AT group. The frequency of the AA genotype was 13% in the healthy normospermic (N) group, 27.4% in the AZ group [OR (95% CI)=2.535], 26.9% in the O group [OR (95% CI) =2.97], 30% in the T group [OR (95% CI)=2.86] and 15.2% in the AT group. The AG genotype was identified in 22% of the N group, 32.9% of the AZ group [OR (95% CI)=1.74], 34.6% of the O group [OR (95% CI)=1.87], 43.3% of the T group [OR (95% CI)=2.71] and 24.2% of the AT group.

**Table 1:** The sperm parameters in the case group


Group	Sperm parameter	Mean ± SD

Azoospermia	Sperm count (10^6^/mL)	0
Oligospermia	Sperm count (10^6^/mL)	6.6 ± 2.3
Asthenoteratospermia	Motility (grades a+b)%/Morphology (% normal forms)	10.7 ± 5.8/4.7 ± 2.8
Teratospermia	Morphology (% normal forms)	6.9 ± 3.4


**Table 2:** The frequencies of alleles and genotypes of the -308 G/A SNP in the TNFα promoter in the azoospermic, teratospermic, Asthenoteratospermic and Oligospermic groups


Genotype-allele/group	Normospermic (%) n=100	Azoospermic (%) n=91	P; OR (95% CI)	Oligospermic (%) n=26	P; OR (95% CI)

AA	13 (13%)	25 (27.4%)	0.018; 2.535 (1.2-5.3)^*^	7 (26.9%)	0.040; 2.97 (1.076-8.22)^*^
AG	22 (22%)	30 (32.9%)	0.010; 1.74 (0.916-3.20)^*^	9 (34.6%)	0.206; 1.87 (0.736-4.787)
GG^*^	65 (65%)	36 (39.5%)	Ref.	10 (38.5%)	Ref.
AA+AG	35 (35%)	55 (60%)	0.001; 2.837 (1.57-5.107)^*^	16 (61%)	0.024; 2.971 (1.22-7.240)^*^
A	48 (24%)	80 (43.9%)	<0.001; 2.484 (1.604-3.845)^*^	23 (44.2%)	0.005; 2.51 (1.32-4.7)^*^
G^**^	152 (76%)	102 (56%)	Ref.	29 (55.8%)	Ref.
		**Teratospermic (%) n=30**		**Asthenoteratospermic (%) n=33**	
AA	13 (13%)	9 (30%)	0.049; 2.86 (1.083-7.599)^*^	5 (15.2%)	0.772; 1.195 (0.392-3.648)
AG	22 (22%)	13 (43.3%)	0.033; 2.71 (1.143-6.428)^*^	8 (24.2%)	0.812; 1.135 (0.449-2.864)
GG^*^	65 (65%)	8 (26.7%)	Ref.	20 (60.6%)	Ref.
AA+AG	35 (35%)	22 (73%)	<0.001, 5.107 (2.061-12.657)	13 (39%)	0.679; 1.207 (0.537-2.714)
A	48 (24%)	31 (51.7%)	<0.001; 3.385 (1.85-6.17)^*^	18 (27.3%)	0.623; 1.18 (0.632-2.23)
G^**^	152 (76%)	29 (48.3%)	Ref.	48 (72.7%)	Ref.


TNFα; Tumor necrosis factor alpha, SNP; Single nucleotide polymorphism, OR; Odds ratios, CI; Confidence interval, *; Significant P<0.05. Ancestral genotypes GG* and alleles G** were taken as reference.

## Discussion

Genetic variation such as SNPs in TNFα promoter region may affect its expression. There are several studies that have investigated the association of *TNFα* SNPs with different diseases such as colorectal cancer, pre-eclampsia, prostate cancer and Crohn’s disease (21, 22). The basic knowledge about the crucial role of TNFα in spermatogenesis is based on the study by Suh et al. (23) in which male TNFα knockout mice showed delayed spermatogenesis, reduced testis weight and sperm count in comparison with wild-type mice.

In this study, we analyzed the TNFα -308 G/A SNP to identify its possible association with sperm abnormality in Iranian males. To the best of our knowledge, this study has not been undertaken in Iranian males. Our findings indicate that this SNP is significantly associated with azoospermia, oligospermia and teratospermia. In other words, this SNP is among many genetic factors that may lead to a decreased count of sperm and abnormal morphology in our cases.

Similar to our study, Tronchon et al. (13) also found a positive association of the TNFα -308 A allele with oligospermia and teratospermia. Zalata et al. (18) also observed an increased frequency of TNFα -308 GG genotypes in fertile males compared with the infertile group in the Egyptian population. In the Indian population, consistently, the frequency of the AA genotype was higher in infertile individuals rather than fertile subjects, and higher level of apoptosis and necrosis levels were observed in infertile males, likely due to increased levels of reactive oxygen species (19). In contrast, Kurz et al. (24) found no association of TNFα -308 C>T and -863 C>A SNPs with sperm abnormalities (asthenozoospermia and oligo-asthenoteratozoospermia) in the Australian population. In the Greek population, Lazaros et al. (25) also found no association between -863 C>A and semen quality. The differences between the results of studies can be related to different number of studied individuals and different studied population with subgroups and ethnicities.

Based on the results of previous studies, TNFα is known to affect spermatogenesis by changing the structure of the blood-testis barrier and apical ectoplasmic specialization of Sertoli cells, which may lead to abnormal spermatogenesis (26). Moreover, it affects the Fas ligand system and germ cell apoptosis which have important roles in the germ cell maturation and normal spermatogenesis (27, 28). Binding of the TNFα molecule with its type 1 receptor activates signaling molecules in the transduction pathway, in which adaptor proteins interact with conserved death domains. The adaptor proteins increase activation of caspase-8 causing the release of cytochrome c from mitochondria. This is followed by the configuration of a high molecular weight complex (apoptotic protease activating factor-1, cytochrome C, and caspase-9) that activates caspase-3 and causes cell death (29, 30). Our study certainly has its own limitations and further association studies with more polymorphisms and individuals may provide more representative data on the association of *TNFα* variation with different sperm abnormalities in the Iranian population.

## Conclusion

Our study shows that there is a positive association between TNFα -308 G/A SNP and different sperm abnormalities in the Iranian population. Given that the A allele leads to increased expression of TNFα, anti-TNFα agents could be a useful treatment for male infertility.
